# Network Perspectives on Infectious Disease Dynamics

**DOI:** 10.1155/2011/146765

**Published:** 2012-01-09

**Authors:** Lauren Ancel Meyers, Ben Kerr, Katia Koelle

**Affiliations:** ^1^Section of Integrative Biology, The University of Texas at Austin, 1 University Station C0930, Austin, TX 78712, USA; ^2^Department of Biology, University of Washington, P. O. Box 351800, Seattle, WA 98195, USA; ^3^Department of Biology, Duke University, P. O. Box 90338, Durham, NC 27708, USA

Over the last two decades, network perspectives have become ubiquitous in the life sciences, ranging from the consideration of microscopic molecular networks to macroscopic social interaction networks. These perspectives have provided new insights into the patterns and processes that we, as scientists, aim to understand. They have also encouraged the development of new mathematical and statistical approaches for rigorous examination of biological networks. This special issue focuses specifically on pioneering network perspectives in the field of infectious disease epidemiology, which have already yielded important new understanding of the dynamics of infectious disease outbreaks in humans and animals.

This special issue begins with an excellent introduction for newcomers to the field of network epidemiology by L. Danon and coauthors. This review gives an accessible overview of the types of networks relevant to the study of epidemiological interactions and methods for characterizing structural properties of networks. It also concisely summarizes new mathematical and simulation-based approaches for determining epidemiological dynamics on networks and statistical approaches for estimating epidemiological quantities for contact networks.

The special issue continues with several research articles that present new methodological developments in the field of network epidemiology. The first of these articles, by E. Kenah and J. C. Cohen, examines “epidemic percolation networks” (EPNs), a mathematical approach to modeling infectious disease transmission in networks that unifies and generalizes several existing mathematical approaches for studying infectious disease models (e.g., branching processes and network models). The article demonstrates the application of the methodology to the practical public health problem of designing effective vaccine priorities. The second article, by E. B. O'Dea and C. Wilke, also addresses the unification of existing approaches. Specifically, the authors posit that the evolutionary dynamics of viruses will be shaped by the contact patterns of the hosts they infect. They quantify the impact of host contact networks on viral gene genealogies and demonstrate the importance of explicitly considering network structure when estimating epidemiological parameters from viral sequence data.

The third research article, by H. Nishiura et al. addresses assortativity in networks, that is, the commonly observed phenomenon that nodes tend to connect to other nodes with similar properties. For example, in social networks, individuals may be most likely to associate with others in the same age group or other sociodemographic categories. The article presents quantitative approaches for analyzing the impacts of assortative mixing patterns on the probability of pandemic emergence. Based on such analyses, the article offers a plausible explanation for the early delay in geographic spread of the 2009 H1N1 influenza pandemic. This is a clear example of a network perspective on infectious disease dynamics providing a more in-depth understanding of observed epidemiological patterns. The last research article, by M. J. Ferrari and coauthors, also illustrates the scientific value of network concepts. Specifically, using a network perspective, the authors are able to reconcile conflicting patterns of density-dependent and frequency-dependent transmission dynamics. They show that, when comparing across populations, intrapopulation density-dependent transmission on networks can result in apparent frequency-dependent transmission dynamics.

The first five articles in the special issue review past work and present new results in network epidemiology. However, the field is quite young, with many exciting developments and applications still to come. Thus, we devote the last third of this special issue to future research. The first article in this section, by M. E. Craft and D. Caillaud, argues that network perspectives have been largely limited to human applications and encourages their use in other systems, particularly wildlife populations. The article describes advances in data collection that must come first and touches on the critical need for iterative interplay between theory and data. This iterative interplay is the major focus of our special issue's last article, by R. Rothenberg and E. Costenbader, which tackles the issue of connecting theory and data to advance network epidemiology. The authors end with a directive for our theoretical colleagues to more closely “snuggle to the facts” to ensure that our modeling efforts translate into meaningful and practical insights into the spread of infectious diseases.

Taken together, we hope that these articles give you, the reader, a taste of the past, present, and future of network epidemiology. Most of all, we hope that this issue whets your appetite and stimulates further interest in developing and harnessing network perspectives on infectious disease dynamics.

